# An increased chloride level in hypochloremia is associated with decreased mortality in patients with severe sepsis or septic shock

**DOI:** 10.1038/s41598-017-16238-z

**Published:** 2017-11-21

**Authors:** Hyung Jung Oh, Seung Jun Kim, Yong Chan Kim, Eun Jin Kim, In Young Jung, Dong Hyun Oh, Su Jin Jeong, Nam Su Ku, Sang Hoon Han, Jun Yong Choi, Young Goo Song, Dong-Ryeol Ryu, June Myung Kim

**Affiliations:** 1 Ewha Institute of Convergence Medicine, Ewha Womans University Mokdong Hospital, Seoul, Korea; 2 Research Institute for Human Health Information, Ewha Womans University Mokdong Hospital, Seoul, Korea; 3 Department of Internal Medicine, International St. Mary’s Hospital, Catholic Kwandong University, College of Medicine, Incheon, Korea; 4 Department of Internal Medicine, Yonsei University, College of Medicine, Seoul, Korea; 5 AIDS research institute, Yonsei University College of Medicine, Seoul, Korea; 6 Department of Internal Medicine, School of Medicine, Ewha Womans University, Seoul, Korea; 7 Tissue Injury Defense Research Center, Ewha Womans University, Seoul, Korea

## Abstract

Only a few observational studies investigated the association between hypochloremia and mortality in critically ill patients, and these studies included small number of septic patients. Also, no study has evaluated the effect of an increase in chloride (Cl^−^) concentration in hypochloremia on the mortality. A total of 843 Korean septic patients were divided into three groups based on their baseline Cl^−^ level, and Cox analyses were performed to evaluate the 28-day mortality. Moreover, the change in Cl^−^ level (ΔCl) from baseline to 24, 48, or 72 hour was determined, and Cox analyses were also conducted to evaluate the relationship of ΔCl with mortality. 301 (35.7%) patients were hypochloremic (Cl^−^ < 97 mEq/L), and 38 (4.5%) patients were hyperchloremic (Cl^−^ > 110 mEq/L). During the follow-up period, 119 (14.1%) patients died. Hypochloremia was significantly associated with an increased mortality after adjusting for several variables, but an 1 mEq/L increase of ΔCl within 24 hour in patients with hypochloremia was significantly related to a decreased mortality. Caution might be required in severe septic patients with hypochloremia considering their increased mortality rate. However, an increased Cl^−^ concentration might decrease the mortality rate of such patients.

## Introduction

Chloride is the most abundant anion in plasma and interstitial fluid, accounting for approximately one-third of plasma tonicity, and it affects acid‒base changes^1,2^. Although chloride is a major electrolyte measured routinely in daily practice, few studies have focused on chloride levels in critically ill patients^3^. Several studies have reported the incidence of chloride alterations and the association thereof with patient outcomes^4–7^.

Intravenous fluid resuscitation to restore the effective circulatory volume is a mainstay of early therapy for severely ill septic patients^7,8^. During the salvage phase of shock, 0.9% saline is typically infused into these patients^9–11^. However, because saline is rich in chloride^12–14^, these patients are susceptible to hyperchloremia during the post-resuscitation phase^14,15^. Moreover, hyperchloremic metabolic acidosis induced by use of chloride-rich solutions is associated with poor outcomes in critically ill patients^5,14,16^, particularly in those with severe sepsis and septic shock^15^. Thus, whether a chloride-liberal or chloride-restricted solution leads to better clinical outcomes in septic patients remains controversial^17–19^.

Hypochloremia is also independently associated with an increased risk of mortality in critically ill patients because of the occurrence of metabolic alkalosis^20–22^. However, only a few observational studies have evaluated the association between hypochloremia and hospital mortality, and they included only a small number of septic patients in the intensive care unit^6,7^. Accordingly, we stratified 843 Korean patients into hypochloremia, normochloremia, and hyperchloremia groups based on their baseline chloride level and assessed the 28-day mortality rate in the three groups. Furthermore, we investigated the effect of an increased chloride level on the 28-day mortality rate.

## Results

### Baseline characteristics

The mean age of the 843 patients was 65.8 ± 14.1 years, and 434 (51.5%) patients were male. The mean body mass index (BMI) was 23.1 ± 6.3 kg/m^2^, and the mean sequential organ failure assessment (SOFA) score, and acute physiology and chronic health evaluation (APACHE) II score were 8.4 ± 3.0 and 16.9 ± 6.7. The mean systolic blood pressure (SBP), diastolic blood pressure (DBP), and mean arterial pressure (MAP) were 84.1 ± 24.3, 53.2 ± 13.3, and 63.5 ± 16.0 mmHg, respectively. Of the 843 patients, 294 (34.9%) had diabetes mellitus, 461 (54.7%) had hypertension, 70 (8.3%) had congestive heart failure, 136 (16.1%) had cerebrovascular accidents, and 100 (11.9%) had a history of coronary arterial disease. Dementia, cancer, chronic pulmonary disease, chronic liver disease, and kidney disease were diagnosed in 5.2%, 33.9%, 14.0%, 10.6%, and 17.0% of the patients, respectively (Table 1).

**Table 1:** Baseline characteristics at the time of ED admission among three groups (hypochloremia, normochloremia, and hyperchloremia)*.

Variables	Total (n = 843, 100%)	Hypochloremia (n = 301, 35.7%)	Normochloremia (n = 504, 59.8%)	Hyperchloremia (n = 38, 4.5%)	p-value
Age, years	65.8 ± 14.1	64.4 ± 13.8	66.4 ± 14.1	69.7 ± 15.3	0.030
Male, n(%)	434 (51.5%)	162 (53.8%)	255 (50.6%)	17 (44.7%)	0.470
BMI, kg/m^2^	23.1 ± 6.3	22.9 ± 6.8	23.2 ± 6.0	23.0 ± 4.1	0.797
SBP, mmHg	84.1 ± 24.3	83.6 ± 24.9	84.7 ± 24.5	80.1 ± 13.8	0.485
DBP, mmHg	53.2 ± 13.3	53.5 ± 13.9	53.0 ± 13.2	53.5 ± 13.3	0.885
MAP, mmHg	63.5 ± 16.0	63.5 ± 16.7	63.6 ± 16.0	62.3 ± 10.5	0.902
SOFA score	8.4 ± 3.0	8.9 ± 3.0	8.0 ± 2.9	9.6 ± 3.1	<0.001
APACHE II score	16.9 ± 6.7	18.7 ± 5.9	15.3 ± 7.1	20.4 ± 10.8	0.003
**Comorbidity disease, n(%)**					
DM	294 (34.9%)	114 (37.9%)	168 (33.3%)	12 (31.6%)	0.387
Hypertension	461 (54.7%)	159 (52.8%)	281 (55.8%)	21 (55.3%)	0.720
CHF	70 (8.3%)	34 (11.3%)	34 (6.7%)	2 (5.3%)	0.061
CVA	136 (16.1%)	42 (14.0%)	85 (16.9%)	9 (23.7%)	0.240
CAD	100 (11.9%)	34 (11.3%)	62 (12.3%)	4 (10.5%)	0.882
Dementia	44 (5.2%)	12 (4.0%)	27 (5.4%)	5 (13.2%)	0.056
Cancer	286 (33.9%)	103 (34.2%)	166 (32.9%)	17 (44.7%)	0.331
Chronic lung disease	118 (14.0%)	39 (13.0%)	74 (14.7%)	5 (13.2%)	0.783
Chronic liver disease	89 (10.6%)	32 (10.6%)	55 (10.9%)	2 (5.3%)	0.550
Chronic kidney disease	143 (17.0%)	51 (16.9%)	86 (17.1%)	6 (15.8%)	0.980
Acute kidney injury, n(%)	425 (50.4%)	176 (58.5%)	224 (44.4%)	25 (65.8%)	0.011
Vasopressor needs, n(%)	776 (92.1%)	276 (91.7%)	465 (92.3%)	35 (92.1%)	0.852
Mechanical ventilation, n(%)	208 (24.7%)	72 (23.9%)	125(24.8%)	11 (28.9%)	0.791
Mean daily fluid balance (L/d)^†^	0.6 ± 1.3	0.5 ± 1.5	0.4 ± 1.4	0.6 ± 1.7	0.304
Mean daily infused 0.9% saline, (L/d)^††^	1.5 ± 0.7	1.5 ± 0.7	1.5 ± 0.7	1.4 ± 0.8	0.636
**Laboratory values**					
WBC, × 10^3^/mm^3^	13.8 ± 10.6	15.1 ± 11.1	13.2 ± 10.5	11.3 ± 6.4	0.013
Hb, g/dL	12.3 ± 7.2	12.5 ± 8.6	12.2 ± 6.4	11.6 ± 2.5	0.705
Platelet, × 10^3^/mm^3^	199.5 ± 147.5	222.0 ± 202.6	186.6 ± 103.9	192.6 ± 95.8	0.004
BUN, mg/dL	34.7 ± 28.3	40.9 ± 36.0	29.7 ± 20.4	51.5 ± 34.5	<0.001
Creatinine, mg/dL	2.1 ± 2.0	2.7 ± 2.6	1.8 ± 1.3	2.7 ± 2.0	0.005
Albumin, g/dL	3.1 ± 0.7	3.0 ± 0.7	3.1 ± 0.7	2.9 ± 0.7	0.028
Total bilirubin, mg/dL	1.3 ± 1.6	1.4 ± 1.6	1.2 ± 1.4	1.3 ± 3.1	0.310
Lactate, mg/dL	4.2 ± 3.5	5.0 ± 4.1	3.7 ± 3.0	4.3 ± 2.9	<0.001
CRP, mg/L	146.6 ± 107.4	174.7 ± 109.9	131.0 ± 100.5	132.6 ± 131.0	<0.001
Sodium, mEq/L	135.1 ± 6.2	130.2 ± 5.4	137.3 ± 3.9	145.6 ± 7.3	<0.001
Potassium, mEq/L	4.2 ± 0.9	4.4 ± 1.0	4.1 ± 0.8	4.3 ± 1.0	<0.001
Chloride, mEq/L	99.4 ± 7.1	92.3 ± 5.0	102.5 ± 3.1	114.6 ± 3.6	<0.001
total CO_2_, mEq/L	17.7 ± 5.2	17.5 ± 6.1	18.2 ± 4.5	13.5 ± 4.0	<0.001
28-day mortality, n(%)	119 (14.1%)	59 (19.6%)	53 (10.5%)	7 (18.4%)	0.001

Data are expressed as mean (with standard deviation) or n (%). Abbreviations; ED, emergency department; BMI, body mass index; SBP, systolic blood pressure; DBP, diastolic blood pressure; MAP, mean arterial pressure; SOFA, sequential organ failure assessment; APACHE, acute physiology and chronic health evaluation; DM, diabetes mellitus; CHF, congestive heart failure; CVA, cerebrovascular accidents; CAD, coronary arterial disease; WBC, white blood cell; Hb, hemoglobin; BUN, blood urea nitrogen; CRP, C-reactive protein. *We investigated baseline demographic and laboratory data based on the time of patients’ ED admission, and stratified these data based on the hypochloremia, normochloremia, and hyperchloremia groups. Hypochloremia; chloride level less than 98 mEq/L at baseline. Normochloremia; chloride level between 98 to 110 mEq/L at baseline. Hyperchloremia; chloride level over 110 mEq/L at baseline. ^†^To quantify 72-hour cumulative fluid balance, we used the following formula: [∑daily (fluid intake (L)−total output (L)], which was defined as “net fluid accumulation for 72 hours”. “Mean daily fluid balance” was calculated as the arithmetic mean of the daily fluid balance from the admission of ED to 72 hours. ^††^According to our policy of the solution for severe sepsis, we usually use 0.9% saline.

Three hundred and one (35.7%) patients were in the hypochloremic group, and 38 (4.5%) were in the hyperchloremic group. The patients in the hyperchloremic group had a significantly higher SOFA score (9.6 ± 3.1 vs. 8.9 ± 3.0 in the hypochloremic group and 8.0 ± 2.9 in the normochloremic group, *P* < 0.001) and APACHE II score (20.4 ± 10.8 vs. 18.7 ± 5.9 in the hypochloremic group and 15.3 ± 7.1 in the normochloremic group, *P* = 0.003), serum blood urea nitrogen (BUN) level, sodium concentration, and chloride concentration compared with the other two groups. Moreover, there were more occurrence of acute kidney injury (AKI) in hypochloremic and hyperchloremic groups compared with normochloremic group, but there were no significant differences among the three groups in vasopressor needs and mechanical ventilation. White blood cell and platelet counts and serum lactate, C-reactive protein (CRP), and potassium levels were highest in the hypochloremic group. Serum albumin and total CO_2_ levels were significantly highest in the normochloremic group. We investigated “Net fluid accumulation for 72 hours” and calculated “Mean daily fluid balance”. Moreover, we examined the type and amount of fluid for 72 hours from the initial admission of emergency department (ED). Table 1 showed that mean daily fluid balance was 0.6 L and daily infused 0.9% saline was 1.5 L. However, there were no significant differences in mean daily fluid balance and daily infused 0.9% saline solution among the three groups (Table 1). In addition, although total infused fluid amount in hyperchloremic group for 48- and 72-hours were significantly increased compared with those in normochloremic group, there were no significant differences in 24-hour total infused fluid amount, 24-, 48-, and 72-hour infused 0.9% saline solution among the tree groups (Supplementary Tables 1 and 2).

### Hypochloremia and 28-day mortality

**Figure 1:**
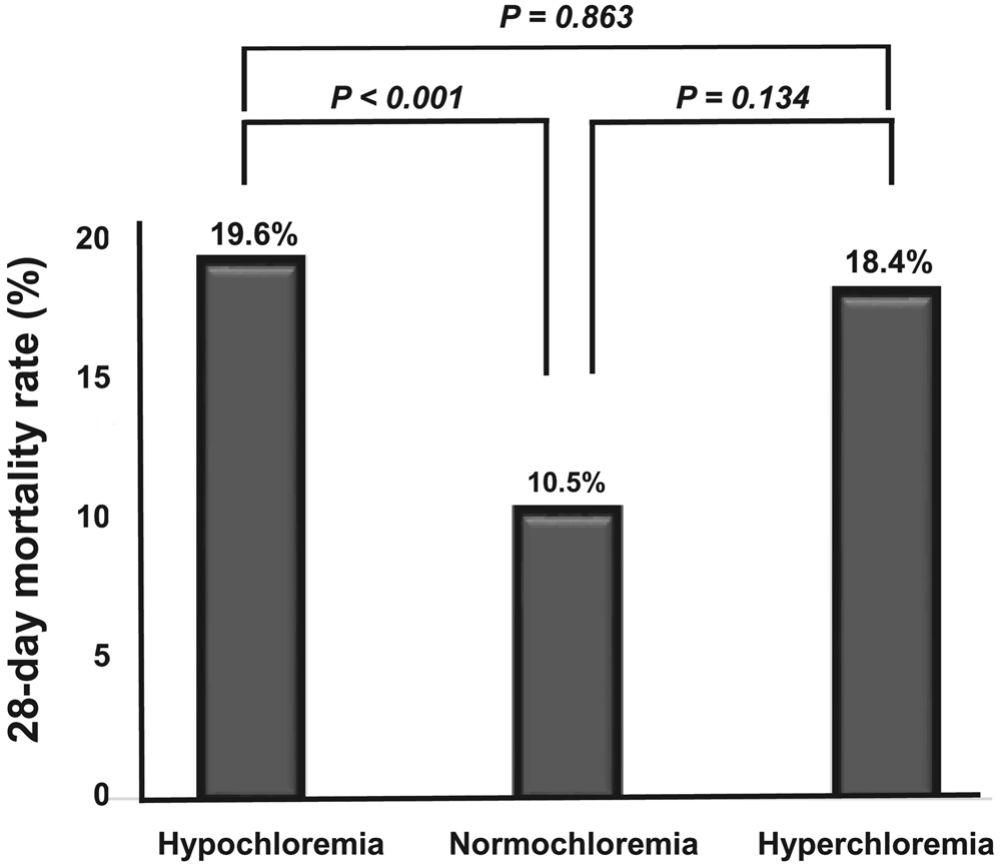
Hypochloremia and 28-day mortality. The 28-day mortality rate was significantly higher in the hypochloremic group (59 patients, 19.6%) compared with the normochloremic group (53 patients, 10.5%), whereas there was no significant difference in 28-day mortality rate between hyperchloremic group and normochloremic group.

**Figure 2:**
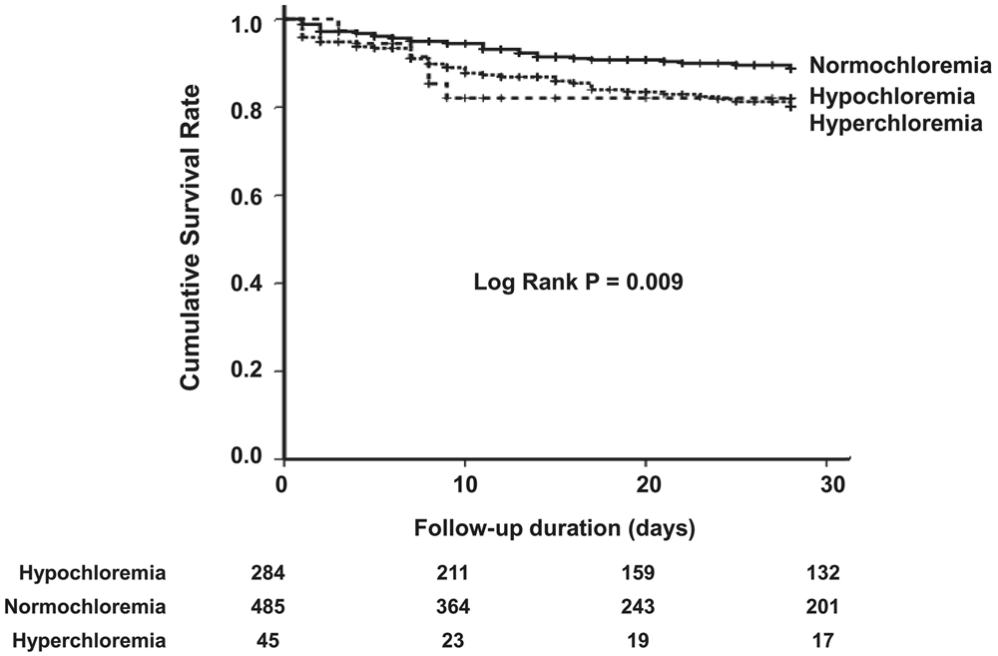
Kaplan-Meier curve for 28-day mortality. The cumulative survival rate wassignificantly lower in the hypochloremia and hyperchloremia groups compared with the normochloremia group (Log rank *P* = 0.009).

One hundred and nineteen (14.1%) patients died during the follow-up period. The 28-day mortality rate was significantly higher in the hypochloremic group (59 patients, 19.6%) and non-significantly higher in the hyperchloremic group (7 patients, 18.4%) compared with the normochloremic group (53 patients, 10.5%) (Table 1 and Fig. 1). In addition, Kaplan-Meier analysis showed that the cumulative survival rate was significantly lower in the hypochloremia and hyperchloremia groups compared with the normochloremia group (Fig. 2). Univariate Cox proportional regression analyses of 28-day mortality revealed that the hypochloremic group had a hazard ratio (HR) of 1.699 [95% confidence interval (CI), 1.129–2.557; *P* = 0.011] compared with the normochloremic group, while no significant association was evident in the hyperchloremic group (HR, 1.762; 95% CI 0.753–4.127; *P* = 0.192). Hypochloremia was also significantly associated with 28-day mortality after adjusting for age, sex, MAP, SOFA score, cerebrovascular accidents, serum BUN, albumin, and lactate compared with normochloremic patients (HR, 1.484; 95% CI, 1.078–2.339, *P* = 0.034) (Table 2).

**Table 2:** Univariate and multivariate Cox proportional hazards analyses for 28-day all-cause mortality.

	Univariate		Multivariate	
	HR (95% CI)	p-value	HR (95% CI)	p-value
Group				
Normochloremia	Reference		Reference	
Hypochloremia	1.699 (1.129–2.557)	0.011	1.484 (1.078–2.339)	0.034
Hyperchloremia	1.762 (0.753–4.127)	0.192	1.354 (0.564–3.250)	0.497
Age (per 1year increase)	1.020 (1.003–1.037)	0.023	1.017 (0.997–1.038)	0.099
Male (vs. Female)	1.274 (0.840–1.932)	0.255	1.147 (0.744–1.768)	0.536
MAP (per 1 mmHg increase)	0.972 (0.956–0.988)	0.001	0.982 (0.966–0.998)	0.030
SOFA score (per 1unit increase)	1.220 (1.147–1.297)	<0.001	1.124 (1.047–1.206)	0.001
CVA (vs. non-CVA)	1.797 (1.120–2.884)	0.015	1.883 (1.129–3.139)	0.015
BUN (per 1 mg/dL increase)	1.010 (1.008–1.013)	<0.001	1.004 (1.001–1.008)	0.016
Albumin (per 1 g/dL increase)	0.369 (0.282–0.483)	<0.001	0.411 (0.302–0.559)	<0.001
Lactate (per 1 mg/dL increase)	1.163 (1.114–1.214)	<0.001	1.115 (1.057–1.175)	<0.001

Abbreviations; HR, hazard ratio; CI, confidence interval; MAP, mean arterial pressure; SOFA, sequential organ failure assessment; CVA, cerebrovascular accidents; BUN, blood urea nitrogen.

### The change of serum chloride concentration and 28-day mortality

The effect of an 1 mEq/L increase in the chloride concentration on the 28-day mortality rate was investigated next. The ΔCl_24h_, ΔCl_48h_, and ΔCl_72h_ values were calculated and subjected to univariate Cox proportional regression analyses to determine their effects on 28-day mortality.

In the hypochloremic group, 28-day mortality was significantly decreased with each 1 mEq/L increase in the chloride concentration at 24 hour (HR, 0.914; 95% CI, 0.866–0.966; *P* = 0.001) and 48 hour (HR, 0.936; 95% CI, 0.888–0.987; *P* = 0.014), while there was no significant effect at 72 hour (HR, 0.961; 95% CI, 0.910–1.014; *P* = 0.258) (Table 3). In the normochloremic group, the 28-day mortality rate was significantly reduced by 12.1%, 9.7%, and 10.5% for each 1 mEq/L increase in the chloride concentration at 24, 48, and 72 hour, respectively (Table 4). However, the ΔCl values were not significantly associated with the 28-day mortality rate at any of the time points in the hyperchloremic group (Table 5). Then, multivariate Cox analyses were performed, when the variables had a significant HRs such as ΔCl_24h_, ΔCl_48h_ in hypochloremic group and ΔCl_24h_, ΔCl_48h_, and △Cl_72h_ in normochloremic group. Tables 6 and 7 revealed that the 28-day mortality rate in the hypochloremic group was significantly decreased by 5.4% at 24 hour with an 1 mEq/L increase in the chloride level, after adjusting for age, sex, BMI, SBP, SOFA score, coronary arterial disease (CAD), serum albumin, and total CO_2_, whereas there was no significant effect of an 1 mEq/L increase in the chloride level on 28-day mortality at 48 hour (Table 6). In addition, in normochloremic group, increase of chloride level was not significantly associated with 28-day mortality in any time (Table 7).

**Table 3:** Univariate Cox proportional hazards analyses for 28-day all-cause mortality per 1 mEq/L increase of chloride concentration in hypochloremic group.

Variables (n = 297)	HR (95% CI)	p-value
Delta chloride for 24 hours (ΔCl_24h_)	0.914 (0.866–0.966)	0.001
Delta chloride for 48 hours (ΔCl_48h_)	0.936 (0.888–0.987)	0.014
Delta chloride for 72 hours (ΔCl_72h_)	0.961 (0.910–1.014)	0.258

Abbreviations; HR, hazard ratio; CI, confidence interval. Definitions; The 24 hour delta chloride concentration (ΔCl_24h_) (mEq/L) was calculated by the following formula: [Cl_24h_] − [Cl_at baseline_], where Cl_24h_ is the chloride concentration at 24 hour, and Cl_at baseline_ is the baseline chloride concentration. The 48 hour delta chloride concentration (ΔCl_48h_) (mEq/L) was calculated by the following formula: [Cl_48h_] − [Cl_at baseline_], where Cl_48h_ is the chloride concentration at 48 hour, and Cl_at baseline_ is the baseline chloride concentration. The 72 hour delta chloride concentration (ΔCl_72h_) (mEq/L) was calculated by the following formula: [Cl_72h_] − [Cl_at baseline_], where Cl_72h_ is the chloride concentration at 72 hour, and Cl_at baseline_ is the baseline chloride concentration.

**Table 4:** Univariate Cox proportional hazards analyses for 28-day all-cause mortality per 1 mEq/L increase of chloride concentration in normochloremic group.

Variables (n = 498)	HR (95% CI)	p-value
Delta chloride for 24 hours (ΔCl_24h_)	0.879 (0.821–0.942)	<0.001
Delta chloride for 48 hours (ΔCl_48h_)	0.903 (0.842–0.967)	0.004
Delta chloride for 72 hours (ΔCl_72h_)	0.895 (0.837–0.957)	0.001

Abbreviations; HR, hazard ratio; CI, confidence interval. Definitions; The 24 hour delta chloride concentration (ΔCl_24h_) (mEq/L) was calculated by the following formula: [Cl_24h_] − [Cl_at baseline_], where Cl_24h_ is the chloride concentration at 24 hour, and Cl_at baseline_ is the baseline chloride concentration. The 48 hour delta chloride concentration (ΔCl_48h_) (mEq/L) was calculated by the following formula: [Cl_48h_] − [Cl_at baseline_], where Cl_48h_ is the chloride concentration at 48 hour, and Cl_at baseline_ is the baseline chloride concentration. The 72 hour delta chloride concentration (ΔCl_72h_) (mEq/L) was calculated by the following formula: [Cl_72h_] − [Cl_at baseline_], where Cl_72h_ is the chloride concentration at 72 hour, and Cl_at baseline_ is the baseline chloride concentration.

**Table 5:** Univariate Cox proportional hazards analyses for 28-day all-cause mortality per 1 mEq/L increase of chloride concentration in hyperchloremic group.

Variables (n = 48)	HR (95% CI)	p-value
Delta chloride for 24 hours (ΔCl_24h_)	1.089 (0.966–1.228)	0.161
Delta chloride for 48 hours (ΔCl_48h_)	0.983 (0.870–1.111)	0.787
Delta chloride for 72 hours (ΔCl_72h_)	0.920 (0.777–1.088)	0.330

Abbreviations; HR, hazard ratio; CI, confidence interval. Definitions; The 24 hour delta chloride concentration (ΔCl_24h_) (mEq/L) was calculated by the following formula: [Cl_24h_] − [Cl_at baseline_], where Cl_24h_ is the chloride concentration at 24 hour, and Cl_at baseline_ is the baseline chloride concentration. The 48 hour delta chloride concentration (ΔCl_48h_) (mEq/L) was calculated by the following formula: [Cl_48h_] − [Cl_at baseline_], where Cl_48h_ is the chloride concentration at 48 hour, and Cl_at baseline_ is the baseline chloride concentration. The 72 hour delta chloride concentration (ΔCl_72h_) (mEq/L) was calculated by the following formula: [Cl_72h_] − [Cl_at baseline_], where Cl_72h_ is the chloride concentration at 72 hour, and Cl_at baseline_ is the baseline chloride concentration.

**Table 6:** Multivariate Cox proportional hazards analyses for 28-day all-cause mortality per increase of chloride concentration for 24 & 48 hours in hypochloremic group.

Variables (n = 297)	Multivariate			
	24 hours		48 hours	
	HR (95% CI)	p-value	HR (95% CI)	p-value
Delta chloride (per 1 mEq/L increase of chloride level)	0.946 (0.888–0.997)	0.023	0.961 (0.907–1.018)	0.174
Age (per 1year increase)	1.033 (0.998–1.069)	0.065	1.039 (1.002–1.078)	0.041
Male (vs. Female)	1.082 (0.542–2.160)	0.823	1.103 (0.530–2.297)	0.793
BMI (per 1 kg/m^2^ increase)	1.028 (1.003–1.054)	0.028	1.034 (1.007–1.060)	0.012
SBP (per 1 mmHg increase)	0.987 (0.971–1.003)	0.107	0.994 (0.978–1.009)	0.435
SOFA score (per 1unit increase)	1.129 (1.014–1.257)	0.026	1.113 (0.992–1.248)	0.067
CAD (vs. non-CAD)	2.165 (0.959–4.891)	0.063	2.279 (0.990–5.248)	0.053
Albumin (per 1 g/dL increase)	0.427 (0.261–0.699)	0.001	0.376 (0.227–0.622)	<0.001
Total CO_2_ (per 1mEq/L increase)	0.925 (0.871–0.982)	0.011	0.912 (0.855–0.972)	0.004

Abbreviations; HR, hazard ratio; CI, confidence interval; BMI, body mass index; SBP, systolic blood pressure; SOFA, sequential organ failure assessment; CAD, coronary arterial disease.

**Table 7:** Multivariate Cox proportional hazards analyses for 28-day all-cause mortality per increase of chloride concentration for 24, 48 & 72 hours in normochloremic group.

Variables (n = 297)	Multivariate					
	24 hours		48 hours		72 hours	
	HR (95% CI)	Variables (n = 297)	HR (95% CI)	Variables (n = 297)	HR (95% CI)	Variables (n = 297)
Delta chloride (per 1 mEq/L increase of chloride level)	0.930 (0.853–1.014)	0.098	0.986 (0.907–1.072)	0.747	0.975 (0.896–1.062)	0.562
Age (per 1year increase)	1.008 (0.979–1.037)	0.604	1.009 (0.977–1.042)	0.576	1.004 (0.972–1.038)	0.792
Male (vs. Female)	1.372 (0.711–2.648)	0.345	1.485 (0.704–3.133)	0.300	1.848 (0.827–4.127)	0.134
SOFA score (per 1unit increase)	1.117 (1.000–1.248)	0.050	1.134 (1.005–1.279)	0.042	1.124 (0.992–1.274)	0.066
BUN (per 1 mg/dL increase)	1.013 (1.003–1.023)	0.008	1.013 (1.001–1.024)	0.028	1.014 (1.002–1.026)	0.027
Albumin (per 1 g/dL increase)	0.527 (0.328–0.845)	0.008	0.334 (0.201–0.556)	<0.001	0.339 (0.200–0.573)	<0.001
Total CO_2_ (per 1mEq/L increase)	0.996 (0.918–1.080)	0.915	1.024 (0.933–1.123)	0.620	1.027 (0.934–1.130)	0.579

Abbreviations; HR, hazard ratio; CI, confidence interval; SOFA, sequential organ failure assessment; BUN, blood urea nitrogen.

## Discussion

The 28-day mortality rate was significantly increased by 48.4% in the hypochloremic group compared with the normochloremic group, while it was not significantly increased in the hyperchloremic group compared with the normochloremic group. In contrast, in the hypochloremic group, the 28-day mortality rate was significantly decreased by 5.4% with an 1 mEq/L increases in ΔCl_24h_.

Although the mechanism is unclear, hypochloremia is considered to be related to mortality in critically ill patients, possibly because of metabolic alkalosis^20–22^. However, the main factor responsible for mortality due to hypochloremia and metabolic alkalosis is unclear^7,23^. Although their study population was different from ours, Tani *et al*.^7^ reported that patients with metabolic acidosis had the highest mortality rate (metabolic acidosis, 17.4%; metabolic alkalosis, 3.9%; normal, 6.27%; *P* = 0.027). Moreover, hypochloremic patients with metabolic acidosis had a higher mortality rate compared with those with metabolic alkalosis, suggesting that hypochloremia is associated with mortality independently of metabolic alkalosis.

Hypochloremia could be a sign of the severity of illness^6^ as a result of dysregulated homeostasis, including the serum chloride concentration. The SOFA score was significantly increased in the hypochloremic group compared with the normochloremic group in the current study. However, the hypochloremic group was independently associated with an increased 28-day mortality rate compared with the normochloremic group even after adjusting for the SOFA score. In addition, an increase in chloride level at 24 hour was significantly related to a decreased 28-day mortality rate in the hypochloremic group.

Demonstrating that hypochloremia is a risk factor for mortality is problematic, because we did not show a causal link. Therefore, our results suggest an association between hypochloremia and a higher 28-day mortality rate in septic patients. The increase in the chloride concentration at 24 hour in the hypochloremic group was significantly associated with a decreased mortality rate.

In contrast, hyperchloremia was not significantly related to an increased 28-day mortality rate in this study. Unlike previous studies, the hyperchloremic group comprised only 38 (4.5%) patients, hampering identification of a significant association between hyperchloremia and an increased 28-day mortality rate. Thus, further studies involving larger populations are warranted to determine the relationship between hyperchloremia and mortality.

Intravenous fluid resuscitation is considered essential for early therapy in severe septic patients^4,7,8^, and chloride-rich solutions are typically used during the salvage phase of shock^5,12–14^. Such patients are susceptible to hyperchloremia during the post-resuscitation phase, and the consequent hyperchloremic metabolic acidosis leads to poor clinical outcomes in critically ill patients^5,14–16^. Therefore, several studies have investigated whether a chloride-liberal or chloride-restricted solution leads to better clinical outcomes in septic patients. Some studies reported a chloride-restricted solution to be superior^18,19^, while others suggested no significant difference in clinical outcomes^17,24,25^. To our knowledge, no study has evaluated the influence of an increased chloride concentration on mortality in hypochloremic septic patients. An increase in the chloride level from baseline during follow-up was associated with all-cause hospital mortality^5^ and the development of AKI^4^; however, these studies did not separate hypochloremic patients from normochloremic patients. Therefore, the analysis of the effect of elevated chloride levels on mortality in hypochloremic, normochloremic, and hyperchloremic patients was a strength of this study. Moreover, we report that an increased chloride level was independently associated with a decreased mortality rate in the hypochloremic group, suggesting that chloride-rich solutions could improve the clinical outcomes of hypochloremic patients.

This study had several limitations. First, this was a retrospective cohort study and thus was subject to selection bias. Second, the patients were arbitrarily classified into three groups according to our hospital reference, and the number of patients was distributed unevenly among the three groups. However, there are no established cut-off values for hypo-, normo-, or hyperchloremia^5–7^; most studies use cut-off values of 98 mEq/L for hypochloremia and 110 mEq/L for hyperchloremia^5,6^. Third, we did not investigate acid‒base changes according to alteration of the chloride level. Also, we analyzed only the change in chloride level within 72 hour; thus, our findings cannot be extrapolated beyond 72 hour. Despite these limitations, this study showed that hypochloremia is independently associated with an increased 28-day mortality rate, and that an increased chloride concentration is independently related to a decreased 28-day mortality rate in hypochloremic patients. In contrast a few previous studies presented hypochloremia is significantly associated with adverse clinical outcomes, they did not provide the effect of change of serum chloride concentration. Moreover, this study may be a cornerstone to prove the importance of chloride fluid replacement in the septic patients who have hypochloremia at baseline.

In conclusion, septic critically ill patients with hypochloremia at baseline should be monitored closely. Moreover, such patients may benefit from an increased chloride level. Therefore, a randomized-controlled interventional study involving a larger population is warranted to evaluate the association between an increased chloride level and the mortality rate of hypochloremic patients.

## Materials and Methods

### Study population

A retrospective cohort study was conducted in Severance Hospital, a 2,400-bed tertiary teaching hospital at Yonsei University College of Medicine, South Korea. This study was approved by the institutional review board (IRB) of the Yonsei University Health System Clinical Trial Center (4-2017-0078). The need for informed consent from patients was waived because of the retrospective design of the study. All clinical investigations were conducted in accordance with the guidelines of the 2013 Declaration of Helsinki.

Data were collected from patients admitted to the ED for severe sepsis or septic shock from January 2010 to December 2015. The exclusion criteria were (a) age <18 years, (b) any contraindication for central venous catheterization, (c) pregnancy, (d) acute CVA e) acute coronary syndrome, (f) active gastrointestinal bleeding, (g) trauma, (h) drug overdose, (i) requirement for immediate surgery, (j) transfer to another institution, and (k) a do-not-resuscitate order. Severe sepsis and septic shock were defined according to standard criteria^26^. All patients were included in the study only once.

### Data collection

Baseline characteristics, including demographic information and pre-existing chronic comorbidities, were collected at the time of admission to the ED. The SOFA^27^ and APACHE II^28^ scores were determined to assess disease severity based on the worst score obtained during the initial 24 hour of ED admission. In addition, the serum chloride concentration was measured by indirect potentiometry (ADVIA 1800 Chemistry System; Siemens Healthcare Diagnostic Inc., Oakville, ON, Canada). Chloride levels were determined at 24, 48, and 72 hour from baseline (time of admission to ED), and the change in chloride level relative to the baseline level was calculated for each time point.

### Definitions

1. Hypochloremia was defined as a chloride concentration of <98 mEq/L at baseline. Normochloremia was defined as a chloride concentration of 98–110 mEq/L at baseline. Hyperchloremia was defined as a chloride concentration of >110 mEq/L at baseline.
2. The 24 hour delta chloride concentration (ΔCl_24h_) (mEq/L) was calculated by the following formula: [Cl_24h_] − [Cl_at baseline_], where Cl_24h_ is the chloride concentration at 24 hour, and Cl_at baseline_ is the baseline chloride concentration.
3. The 48 hour delta chloride concentration (ΔCl_48h_) (mEq/L) was calculated by the following formula: [Cl_48h_] − [Cl_at baseline_], where Cl_48h_ is the chloride concentration at 48 hour, and Cl_at baseline_ is the baseline chloride concentration.
4. The 72 hour delta chloride concentration (ΔCl_72h_) (mEq/L) was calculated by the following formula: [Cl_72h_] − [Cl_at baseline_], where Cl_72h_ is the chloride concentration at 72 hour, and Cl_at baseline_ is the baseline chloride concentration.
5. AKI is defined as any of the followings; Increase in serum creatinine by ≥0.3 mg/dL within 48 hours or increase in serum creatinine to ≥1.5 times baseline, which is known or presumed to have occurred within the prior 7 days or urine volume <0.5 mL/kg/h for 6 hours^29^.
6. Net fluid accumulation was measured by the formula: [∑daily (fluid intake (L) − total output (L)]^30^.
7. Mean daily fluid balance was also calculated as the arithmetic mean of the daily fluid balance from the admission of ED to 72 hours^31^.
8. According to our policy of the solution for severe sepsis, we usually use 0.9% saline.

### Study outcomes

The observation period ended 28 days after ED admission, and the primary outcome was the 28-day all-cause mortality rate.

### Statistical analysis

Statistical analysis was performed using SPSS for Windows, version 22.0 (SPSS Inc., Chicago, IL, USA). The patients were classified into hypochloremia, normochloremia, and hyperchloremia groups based on their baseline chloride levels. Continuous variables are presented as means ± standard deviation and categorical variables as numbers and percentages. The baseline characteristics of the groups were compared by analysis of variance for continuous variables and the χ^2^ test for categorical variables.

Kaplan-Meier survival curves were assessed using the log-rank test to compare the differences in 28-day mortality among groups. A Cox model was fit to evaluate the influence of the chloride level on 28-day mortality and the effect of an increased chloride level at 24, 48, or 72 hour from baseline on 28-day mortality. First, we selected variables using the threshold of *P*-value < 0.2 from univariate analysis (Supplementary Table 3), and then confirmed new variables for adjustment of multivariate Cox analysis after assessing colinearity between the selected variables. Finally, MAP, SOFA score, CVA, serum BUN, albumin, and lactate were chosen to adjust for multivariate Cox analysis including age and sex to evaluate the effect of hypochloremia or hyperchloremia on 28-day mortality. With the same way, we performed multivariate Cox analyses for the effects of increase of chloride level on the mortality; BMI, SBP, SOFA score, CAD, serum albumin and total CO_2_ were selected for adjustment including age and sex in hypochloremic group (Table 6), and SOFA score, serum BUN, albumin, and total CO_2_ were adjusted for multivariate Cox analysis including age and sex in normochloremic group (Table 7). The results are presented as hazard ratios (HRs) with 95% confidence intervals (CIs). To confirm the assumption of proportionality, a time-dependent covariate analysis was performed, the results of which were not statistically significant, suggesting that the proportional hazards assumption was reasonable. All tests were two-sided, and a *P* value of <0.05 was considered to indicate statistical significance.

## Electronic supplementary material

Supplementary tables
