# Supplementary material for: An increased chloride level in hypochloremia is associated with decreased mortality in patients with severe sepsis or septic shock
Source: Sci Rep. 2017 Nov 21;7:15883. doi: 10.1038/s41598-017-16238-z (PMC5698440; doi:10.1038/s41598-017-16238-z)
Supplement: Supplementary file 1 — Supplementary tables [file 41598_2017_16238_MOESM1_ESM.doc]

**An increased chloride level in hypochloremia is associated with decreased mortality in patients with severe sepsis or septic shock**

Hyung Jung Oh^1,2†^, Seung Jun Kim^3†^, Yong Chan Kim^4,5^, Eun Jin Kim^4,5^, In Young Jung^4,5^, Dong Hyun Oh^4,5^ Su Jin Jeong^4,5^, Nam Su Ku^4,5^*, Sang Hoon Han^4,5^, Jun Yong Choi^4,5^, Young Goo Song^4,5^, Dong-Ryeol Ryu^2,6,7^, and June Myung Kim^4,5^

^1^Ewha Institute of Convergence Medicine and ^2^Research Institute for Human Health Information, Ewha Womans University Mokdong Hospital;

^3^Department of Internal Medicine, International St. Mary’s Hospital, Catholic Kwandong University College of Medicine, Incheon, Korea

^4^Department of Internal Medicine, Yonsei University College of Medicine, Seoul, Korea

^5^AIDS research institute, Yonsei University College of Medicine, Seoul, Korea

^6^Department of Internal Medicine, School of Medicine and ^7^Tissue Injury Defense Research Center, Ewha Womans University, Seoul, Korea

**Running title:** Hypochloremia in severe sepsis/septic shock

*Correspondence to:

Nam Su Ku, MD.

Department of Internal Medicine

Yonsei University College of Medicine

50-1 Yonsei-ro, Seodaemun-gu

120-752 Seoul

Republic of Korea

Tel.: +82-2-2227-2288

Fax: +82-2-2393-6884

E-mail: [smileboy9@yuhs.ac](mailto:smileboy9@yuhs.ac)

†Oh HJ and Kim SJ contributed equally to this manuscript.

**Supplementary Table 1. Total infused fluid amount in each follow-up duration**

|  | 0 to 24 hours total fluid (L) | 0 to 48 hours total fluid (L)^*^ | 0 to 72 hours total fluid (L)^*^ |
| --- | --- | --- | --- |
| Hypochloremia | 3.2±1.8 | 5.6±2.5 | 7.6±3.2 |
| Normochloremia | 3.0±1.3 | 5.3±2.0 | 7.3±2.7 |
| Hyperchloremia | 3.3±1.6 | 6.2±2.3 | 8.6±3.1 |

Abbreviations; ED, emergent department

Hypochloremia; chloride level less than 98 mEq/L at baseline

Normochloremia; chloride level between 98 to 110 mEq/L at baseline

Hyperchloremia; chloride level over 110 mEq/L at baseline

*P < 0.05

**Supplementary Table 2(A). Total infused fluid amount for 24 hours with each solution**

|  | 0.9% Saline (L) | TPN (L) | Sugar solution (L)^*^ | Packed RBC (mL) | 20% Albumin (mL)^*^ |
| --- | --- | --- | --- | --- | --- |
| Hypochloremia | 2.2±1.3 | 0.6±0.7 | 0.4±0.7 | 61.4±265 | 21.2±49.9 |
| Normochloremia | 2.0±1.1 | 0.6±0.7 | 0.3±0.5 | 36.0±171 | 13.0±39.6 |
| Hyperchloremia | 2.2±1.2 | 0.5±0.7 | 0.5±0.7 | 103±364 | 21.1±47.4 |

Abbreviations; ED, emergent department; TPN, total parenteral nutrition solution; RBC, red blood cell

The composition of TPN is 500 mL of 50% dextrose and 500 mL of 10% amino acid.

Sugar solution is 5% or 10% dextrose solution.

The component of 20% albumin is 200g/L of human albumin, 48 to 100 mmol/L of sodium, and 32 mmol/L of octanoate.

Hypochloremia; chloride level less than 98 mEq/L at baseline

Normochloremia; chloride level between 98 to 110 mEq/L at baseline

Hyperchloremia; chloride level over 110 mEq/L at baseline

*P < 0.05

Supplementary Table 2(B). Total infused fluid amount for 48 hours with each solution

|  | 0.9% Saline (L) | TPN (L) | Sugar solution (L)^‡^ | Packed RBC (mL) | 20% Albumin (mL)^*^ |
| --- | --- | --- | --- | --- | --- |
| Hypochloremia | 3.4±1.7 | 1.3±1.1 | 0.7±1.1 | 103±393 | 50.3±79.0 |
| Normochloremia | 3.3±1.5 | 1.4±1.1 | 0.5±0.8 | 61.6±232 | 35.4±79.0 |
| Hyperchloremia | 3.3±1.9 | 1.2±1.1 | 1.5±1.2 | 122±377 | 59.5±79.8 |

Abbreviations; ED, emergent department; TPN, total parenteral nutrition solution; RBC, red blood cell

The composition of TPN is 500 mL of 50% dextrose and 500 mL of 10% amino acid.

Sugar solution is 5% or 10% dextrose solution.

The component of 20% albumin is 200g/L of human albumin, 48 to 100 mmol/L of sodium, and 32 mmol/L of octanoate.

Hypochloremia; chloride level less than 98 mEq/L at baseline

Normochloremia; chloride level between 98 to 110 mEq/L at baseline

Hyperchloremia; chloride level over 110 mEq/L at baseline

*P < 0.05; ‡P < 0.001

Supplementary Table 2(C). Total infused fluid amount for 72 hours with each solution

|  | 0.9% Saline (L) | TPN (L) | Sugar solution (L)^‡^ | Packed RBC (mL) | 20% Albumin (mL)^*^ |
| --- | --- | --- | --- | --- | --- |
| Hypochloremia | 4.5±2.2 | 1.9±1.5 | 0.9±1.2 | 141±457 | 72.1±105 |
| Normochloremia | 4.4±2.0 | 2.0±1.5 | 0.7±1.0 | 85.7±273 | 57.6±104 |
| Hyperchloremia | 4.3±2.5 | 2.0±1.5 | 2.1±1.6 | 154±503 | 91.9±112 |

Abbreviations; ED, emergent department; TPN, total parenteral nutrition solution; RBC, red blood cell

The composition of TPN is 500 mL of 50% dextrose and 500 mL of 10% amino acid.

Sugar solution is 5% or 10% dextrose solution.

The component of 20% albumin is 200g/L of human albumin, 48 to 100 mmol/L of sodium, and 32 mmol/L of octanoate.

Hypochloremia; chloride level less than 98 mEq/L at baseline

Normochloremia; chloride level between 98 to 110 mEq/L at baseline

Hyperchloremia; chloride level over 110 mEq/L at baseline

*P < 0.05; ‡P < 0.001

**Supplementary Table 3.** Univariate Cox analysis for 28-day all-cause mortality

| Variables | | HR (95% CI) | p-value |
| --- | --- | --- | --- |
| Age (per 1year increase) | | 1.020 (1.003-1.037) | 0.023 |
| Male (vs. Female) | | 1.274 (0.840-1.932) | 0.255 |
| BMI (per 1kg/m^2^ increase) | | 1.016 (0.998-1.034) | 0.088 |
| SBP (per 1mmHg increase) | | 0.983 (0.971-0.994) | 0.003 |
| DBP (per 1mmHg increase) | | 0.969 (0.951-0.988) | 0.001 |
| MAP (per 1mmHg increase) | | 0.972 (0.956-0.988) | 0.001 |
| SOFA (per 1unit increase) | | 1.220 (1.147-1.297) | <0.001 |
| APACHE II (per 1 unit increase) | | 1.180 (1.077-1.824) | 0.002 |
| DM (vs. non-DM) | | 1.198 (0.787-1.824) | 0.399 |
| Hypertension (vs. non-hypertension) | | 1.149 (0.758-1.740) | 0.513 |
| CHF (vs. non-CHF) | | 1.458 (0.776-2.740) | 0.241 |
| CVA (vs. non-CVA) | | 1.797 (1.120-2.884) | 0.015 |
| CAD (vs. non-CAD) | | 1.475 (0.848-2.566) | 0.169 |
| Dementia (vs. non-dementia) | | 2.159 (1.085-4.299) | 0.028 |
| Cancer (vs. non-cancer) | | 0.993 (0.646-1.528) | 0.976 |
| Chronic lung disease (vs. non-chronic lung disease) | | 0.923 (0.503-1.695) | 0.797 |
| Chronic liver disease (vs. non-chronic liver disease) | | 0.821 (0.397-1.696) | 0.594 |
| Chronic kidney disease (vs. non-chronic kidney disease) | | 0.932 (0.535-1.621) | 0.802 |
| AKI (vs. non-AKI) | | 2.430 (1.557-3.793) | <0.001 |
| WBC (per 1000/mm^3^ increase) | | 1.023 (1.010-1.036) | <0.001 |
| Hb (per 1g/dL increase) | | 1.004 (0.978-1.031) | 0.754 |
| Platelet (per 1000/mm^3^ increase) | | 0.998 (0.996-1.000) | 0.018 |
| BUN (per 1mg/dL increase) | | 1.010 (1.008-1.013) | <0.001 |
| Creatinine (per 1mg/dL increase) | | 1.143 (1.068-1.224) | <0.001 |
| Albumin (per 1g/dL increase) | | 0.369 (0.282-0.483) | <0.001 |
| T. bilirubin (per 1mg/dL increase) | | 1.091 (1.003-1.186) | 0.043 |
| Lactate (per 1mg/dL increase) | | 1.163 (1.114-1.214) | <0.001 |
| CRP (per 1mg/L increase) | | 1.002 (1.001-1.004) | 0.007 |
| Sodium (per 1mEq/L increase) | | 1.003 (0.970-1.037) | 0.878 |
| Potassium (per 1mEq/L increase) | | 1.365 (1.132-1.647) | 0.001 |
| Chloride (per 1mEq/L increase) | | 0.987 (0.959-1.015) | 0.347 |
| Grouping | |  |  |
|  | Normochloremia | Reference |  |
|  | Hypochloremia | 1.699 (1.129-2.557) | 0.011 |
|  | Hyperchloremia | 1.762 (0.753-4.127) | 0.192 |
| Total CO_2_ (per 1mEq/L increase) | | 0.898 (0.864-0.932) | <0.001 |

Abbreviations; BMI, body mass index; SBP, systolic blood pressure; DBP, diastolic blood pressure; MAP, mean arterial pressure; SOFA, sequential organ failure assessment; APACHE, acute physiology and chronic health evaluation; DM, diabetes mellitus; CHF, congestive heart failure; CVA, cerebrovascular accidents; CAD, coronary arterial disease; AKI, acute kidney injury; WBC, white blood cell; Hb, hemoglobin; BUN, blood urea nitrogen; T. bilirubin, total bilirubin; CRP, C-reactive protein

**Supplementary Table 4.** Univariate Cox analysis for 28-day all-cause mortality in hypochloremia at baseline

| Variables | HR (95% CI) | P-value |
| --- | --- | --- |
| Age (per 1year increase) | 1.014 (0.990-1.039) | 0.261 |
| Male (vs. Female) | 1.039 (0.574-1.881) | 0.899 |
| BMI (per 1kg/m^2^ increase) | 1.028 (1.012-1.045) | 0.001 |
| SBP (per 1mmHg increase) | 0.982 (0.967-0.998) | 0.024 |
| DBP (per 1mmHg increase) | 0.965 (0.939-0.991) | 0.009 |
| MAP (per 1mmHg increase) | 0.970 (0.948-0.992) | 0.009 |
| SOFA (per 1unit increase) | 1.202 (1.094-1.320) | <0.001 |
| APACHE II (per 1 unit increase) | 1.098 (1.004-1.547) | 0.004 |
| DM (vs. non-DM) | 1.256 (0.692-2.282) | 0.454 |
| Hypertension (vs. non-hypertension) | 0.985 (0.545-1.780) | 0.960 |
| CHF (vs. non-CHF) | 1.214 (0.513-2.872) | 0.659 |
| CVA (vs. non-CVA) | 2.136 (1.080-4.228) | 0.029 |
| CAD (vs. non-CAD) | 2.434 (1.202-4.928) | 0.013 |
| Dementia (vs. non-dementia) | 2.298 (0.822-6.426) | 0.113 |
| Cancer (vs. non-cancer) | 0.875 (0.469-1.632) | 0.674 |
| Chronic lung disease (vs. non-chronic lung disease) | 0.976 (0.385-2.478) | 0.960 |
| Chronic liver disease (vs. non-chronic liver disease) | 0.816 (0.292-2.281) | 0.698 |
| Chronic kidney disease (vs. non-chronic kidney disease) | 0.838 (0.373-1.880) | 0.668 |
| AKI (vs. non-AKI) | 2.012 (1.053-3.846) | 0.034 |
| WBC (per 1000/mm^3^ increase) | 1.016 (0.992-1.041) | 0.195 |
| Hb (per 1g/dL increase) | 1.019 (0.994-1.044) | 0.134 |
| Platelet (per 1000/mm^3^ increase) | 0.998 (0.995-1.000) | 0.063 |
| BUN (per 1mg/dL increase) | 1.008 (1.004-1.012) | <0.001 |
| Creatinine (per 1mg/dL increase) | 1.080 (0.988-1.180) | 0.091 |
| Albumin (per 1g/dL increase) | 0.416 (0.288-0.600) | <0.001 |
| T. bilirubin (per 1mg/dL increase) | 1.183 (1.035-1.353) | 0.014 |
| Lactate (per 1mg/dL increase) | 1.128 (1.065-1.195) | <0.001 |
| CRP (per 1mg/L increase) | 1.002 (0.999-1.004) | 0.212 |
| Sodium (per 1mEq/L increase) | 1.009 (0.954-1.066) | 0.762 |
| Potassium (per 1mEq/L increase) | 1.292 (1.019-1.637) | 0.034 |
| Chloride (per 1mEq/L increase) | 1.010 (0.951-1.073) | 0.739 |
| Total CO_2_ (per 1mEq/L increase) | 0.915 (0.873-0.959) | <0.001 |

Abbreviations; BMI, body mass index; SBP, systolic blood pressure; DBP, diastolic blood pressure; MAP, mean arterial pressure; SOFA, sequential organ failure assessment; APACHE, acute physiology and chronic health evaluation; DM, diabetes mellitus; CHF, congestive heart failure; CVA, cerebrovascular accidents; CAD, coronary arterial disease; AKI, acute kidney injury; WBC, white blood cell; Hb, hemoglobin; BUN, blood urea nitrogen; T. bilirubin, total bilirubin; CRP, C-reactive protein

**Supplementary Table 5.** Univariate Cox analysis for 28-day all-cause mortality in normochloremia at baseline

| Variables | HR (95% CI) | P-value |
| --- | --- | --- |
| Age (per 1year increase) | 1.021 (0.996-1.047) | 0.104 |
| Male (vs. Female) | 1.313 (0.705-2.444) | 0.391 |
| BMI (per 1kg/m^2^ increase) | 0.982 (0.917-1.051) | 0.600 |
| SBP (per 1mmHg increase) | 0.987 (0.971-1.004) | 0.126 |
| DBP (per 1mmHg increase) | 0.971 (0.944-0.999) | 0.044 |
| MAP (per 1mmHg increase) | 0.976 (0.952-1.001) | 0.058 |
| SOFA (per 1unit increase) | 1.234 (1.130-1.347) | <0.001 |
| APACHE II (per 1 unit increase) | 1.109 (1.051-1.438) | 0.011 |
| DM (vs. non-DM) | 1.097 (0.581-2.071) | 0.775 |
| Hypertension (vs. non-hypertension) | 1.312 (0.700-2.457) | 0.397 |
| CHF (vs. non-CHF) | 1.854 (0.728-4.726) | 0.196 |
| CVA (vs. non-CVA) | 1.396 (0.666-2.925) | 0.376 |
| CAD (vs. non-CAD) | 1.001 (0.393-2.552) | 0.998 |
| Dementia (vs. non-dementia) | 1.612 (0.497-5.229) | 0.426 |
| Cancer (vs. non-cancer) | 1.154 (0.611-2.179) | 0.659 |
| Chronic lung disease (vs. non-chronic lung disease) | 0.935 (0.393-2.224) | 0.880 |
| Chronic liver disease (vs. non-chronic liver disease) | 0.936 (0.334-2.627) | 0.900 |
| Chronic kidney disease (vs. non-chronic kidney disease) | 1.211 (0.559-2.622) | 0.627 |
| AKI (vs. non-AKI) | 2.520 (1.321-4.806) | 0.005 |
| WBC (per 1000/mm^3^ increase) | 1.027 (1.011-1.043) | 0.001 |
| Hb (per 1g/dL increase) | 0.943 (0.829-1.073) | 0.374 |
| Platelet (per 1000/mm^3^ increase) | 0.996 (0.992-0.999) | 0.017 |
| BUN (per 1mg/dL increase) | 1.020 (1.012-1.028) | <0.001 |
| Creatinine (per 1mg/dL increase) | 1.245 (1.089-1.423) | 0.001 |
| Albumin (per 1g/dL increase) | 0.359 (0.235-0.548) | <0.001 |
| T. bilirubin (per 1mg/dL increase) | 1.105 (0.952-1.284) | 0.188 |
| Lactate (per 1mg/dL increase) | 1.184 (1.098-1.277) | <0.001 |
| CRP (per 1mg/L increase) | 1.002 (0.999-1.004) | 0.253 |
| Sodium (per 1mEq/L increase) | 1.044 (0.963-1.131) | 0.299 |
| Potassium (per 1mEq/L increase) | 1.457 (1.031-2.058) | 0.033 |
| Chloride (per 1mEq/L increase) | 1.049 (0.953-1.155) | 0.327 |
| Total CO_2_ (per 1mEq/L increase) | 0.893 (0.836-0.955) | 0.001 |

Abbreviations; BMI, body mass index; SBP, systolic blood pressure; DBP, diastolic blood pressure; MAP, mean arterial pressure; SOFA, sequential organ failure assessment; APACHE, acute physiology and chronic health evaluation; DM, diabetes mellitus; CHF, congestive heart failure; CVA, cerebrovascular accidents; CAD, coronary arterial disease; AKI, acute kidney injury; WBC, white blood cell; Hb, hemoglobin; BUN, blood urea nitrogen; T. bilirubin, total bilirubin; CRP, C-reactive protein
